# Supplementary material for: A SMARTR workflow for multi-ensemble atlas mapping and brain-wide network analysis
Source: bioRxiv. 2024 Jul 16:2024.07.12.603299. Preprint. [Version 1] doi: 10.1101/2024.07.12.603299 (PMC11275872; doi:10.1101/2024.07.12.603299)
Supplement: Supplementary file 1 [file NIHPP2024.07.12.603299V1-supplement-1.pdf]

bioRxiv preprint doi: https://doi.org/10.1101/2024.07.12.603299; this version posted July 16, 2024. The copyright holder for this preprint (which was not certified by peer review) is the author/funder, who has granted bioRxiv a license to display the preprint in perpetuity. It is made available under aCC-BY 4.0 International license.

SUPPLEMENTAL INFORMATION

A SMARTR workflow for multi-ensemble atlas mapping and brain-wide network analysis

Michelle Jin[1,2], Simon O. Ogundare[3], Marcos Lanio[1], Sophia Sorid[3], Alicia R. Whye[3], Sofia Leal Santos[4],

Alessandra Franceschini[5], and Christine. A. Denny[5,6]

bioRxiv preprint doi: https://doi.org/10.1101/2024.07.12.603299; this version posted July 16, 2024. The copyright holder for this preprint (which was not certified by peer review) is the author/funder, who has granted bioRxiv a license to display the preprint in perpetuity. It is made available under aCC-BY 4.0 International license.

SUPPLEMENTAL FIGURES AND FIGURE LEGENDS

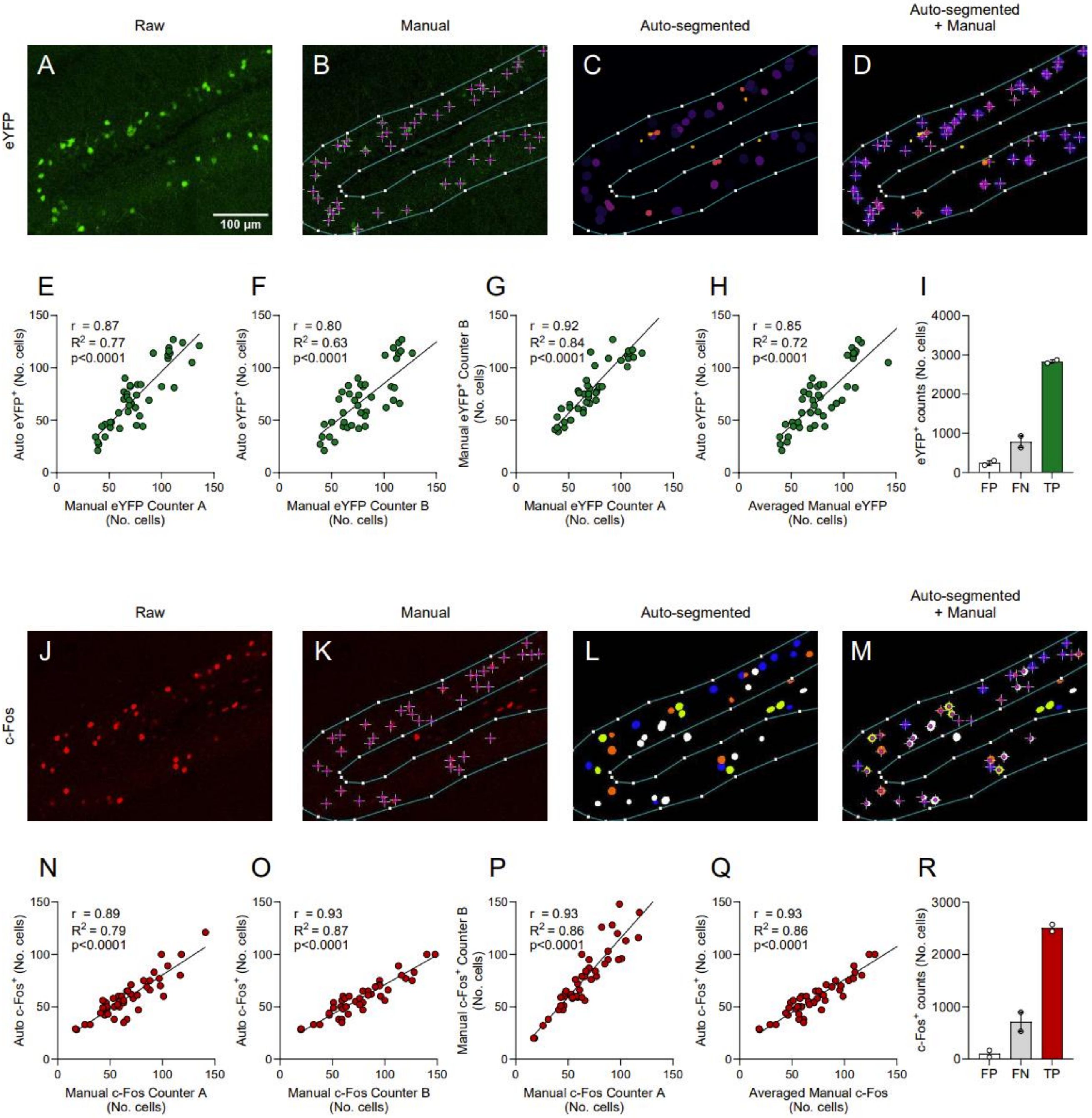

Figure S1. Automated segmentation yields comparable results to manual cell counting. (A) A representative image of the raw eYFP immunolabelled signal (channel 1) in the dentate gyrus (DG). **(B)** An ROI was drawn around the granule cell layer of the dentate gyrus and eYFP$^+$ cells within the boundaries of the ROI were manually counted. **(C)** Detected 3D objects representing eYFP$^+$ cells that were automatically segmented from the raw representative image. **(D)** Qualitative overlay of the manual eYFP$^+$ cell counts and the auto-segmented objects. **(E)** A high correlation (R = 0.87) was found between manual eYFP$^+$ cell counts by

bioRxiv preprint doi: https://doi.org/10.1101/2024.07.12.603299; this version posted July 16, 2024. The copyright holder for this preprint (which was not certified by peer review) is the author/funder, who has granted bioRxiv a license to display the preprint in perpetuity. It is made available under aCC-BY 4.0 International license.

Counter A and the number of auto-segmented objects. Each dot represents one ROI counted. (**F**) A high correlation (R = 0.80) was found between manual $eYFP^+$ cell counts by Counter B and the number of auto-segmented objects. **(G)** There was high inter-rater correlation (R = 0.92) between manual $eYFP^+$ cell counts. **(H)** Averaged manual eYFP+ cell counts showed overall high correlation (R = 0.85) with auto-segmented counts. **(I)** Counts of all false positive (FP), false negative (FN), and true positive (TP) cell counts when comparing auto-segmented $eYFP^+$ counts and manual counts. **(J)** Representative image of the raw c-Fos immunolabelling signal (channel 2) in the DG. **(K)** Manual c-Fos^+ cell counts within the drawn ROI. **(L)** Automatically segmented 3D objects representing c-Fos^+ cells. **(M)** Overlay of manual and auto-segmented c-Fos^+ counts. **(N)** A high correlation (R = 0.89) was found between manual c-Fos^+ cell counts by Counter A and the number of auto-segmented objects. Each dot represents one ROI counted. **(O)** A high correlation (R = 0.93) was found between manual c-Fos^+ cell counts by Counter B and the number of auto-segmented objects. **(P)** There was high inter-rater correlation (R = 0.93) between manual c-Fos^+ cell counts. **(Q)** Averaged manual c-Fos^+ cell counts showed overall high correlation (R = 0.93) with auto-segmented counts. **(R)** Counts of all FP, FN, and TP cell counts when comparing auto-segmented c-Fos^+ counts and manual counts.

bioRxiv preprint doi: https://doi.org/10.1101/2024.07.12.603299; this version posted July 16, 2024. The copyright holder for this preprint (which was not certified by peer review) is the author/funder, who has granted bioRxiv a license to display the preprint in perpetuity. It is made available under aCC-BY 4.0 International license.

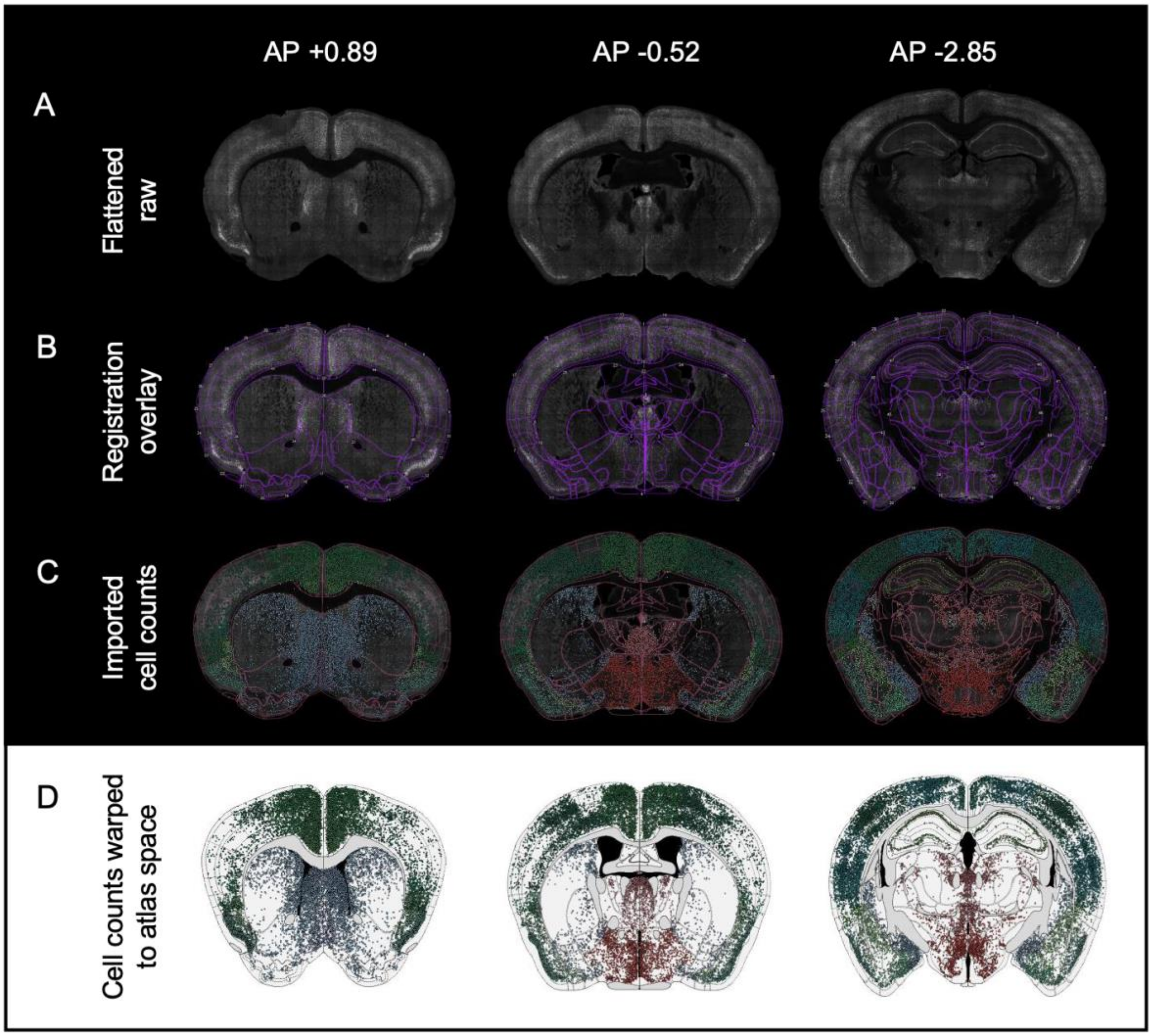

Figure S2. Registration projecting segmented cell counts to a standard atlas space. (A) Raw grayscale images of whole coronal sections with eYFP$^+$ and c-Fos$^+$ signals combined (Flattened raw). Corresponding coordinates of the best matching Allen Mouse Brain atlas plates (AP +0.89, left; AP -0.52, middle; AP -2.85, right) are displayed above. **(B)** The user-corrected registration overlay across the representative coronal sections. User-correction through an interactive console interface was performed by interfacing with the Wholebrain and SMART packages through SMARTR. **(C)** Representative images of imported c-Fos automated cell counts onto the user-corrected registrations (image space). Cells are color coded based on region colors consistent with the Allen Mouse Brain Atlas. **(D)** Forward warp of the segmented c-Fos$^+$ counts from image space to a common shared stereotaxic coordinate space (atlas space) facilitates standardized integration of data across all mice.

bioRxiv preprint doi: https://doi.org/10.1101/2024.07.12.603299; this version posted July 16, 2024. The copyright holder for this preprint (which was not certified by peer review) is the author/funder, who has granted bioRxiv a license to display the preprint in perpetuity. It is made available under aCC-BY 4.0 International license.

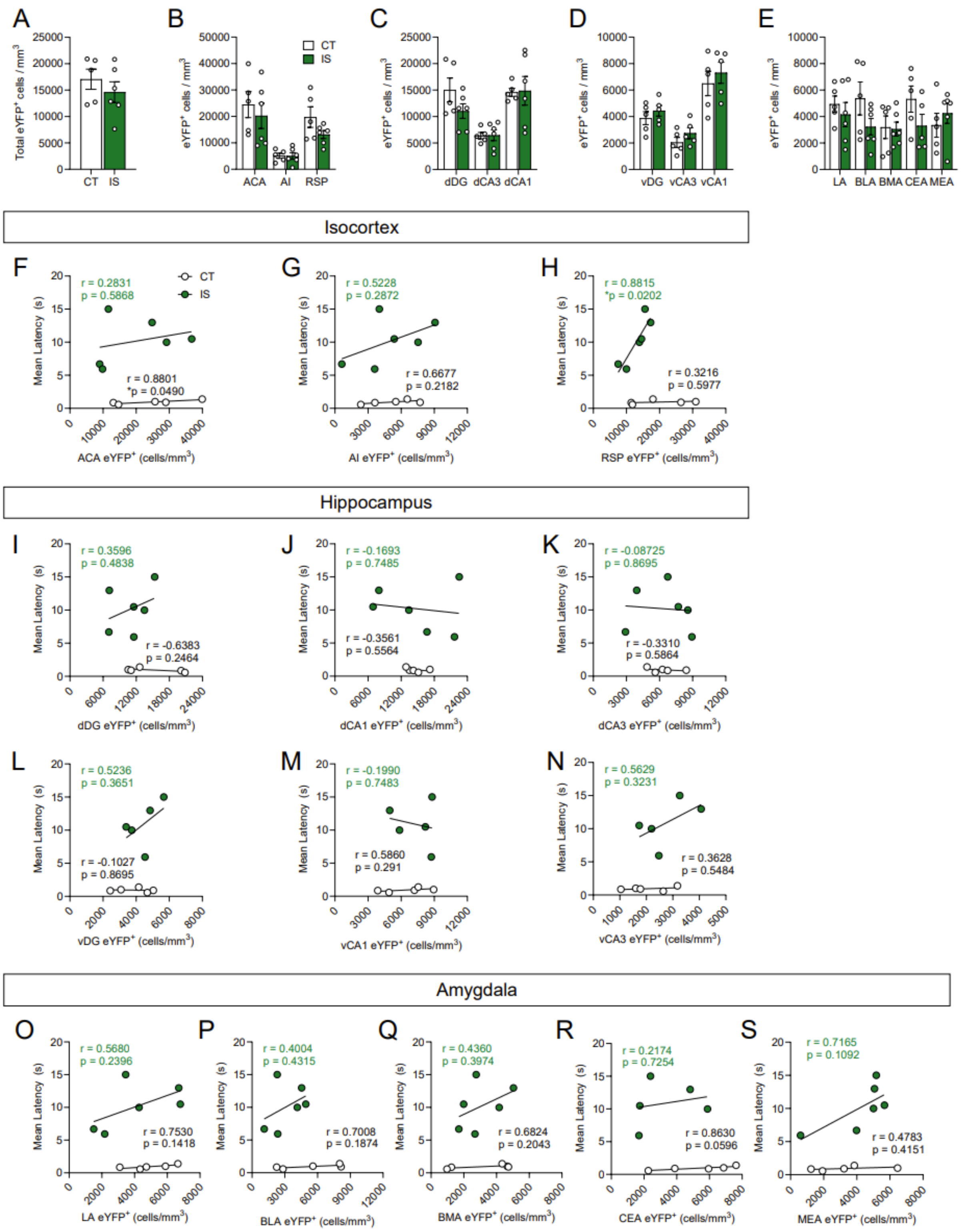

Figure S3. Activity is not heightened globally or across targeted subregions following exposure to inescapable shock. (A) No significant differences were found in total brain-wide volume normalized (cells/mm^3) counts of eYFP$^+$ cells between context trained (CT) or inescapable shock (IS) groups. Regional activation analysis of targeted **(B)** isocortical areas (ACA, AI, RSP), **(C)** dorsal hippocampal regions (dDG,

bioRxiv preprint doi: https://doi.org/10.1101/2024.07.12.603299; this version posted July 16, 2024. The copyright holder for this preprint (which was not certified by peer review) is the author/funder, who has granted bioRxiv a license to display the preprint in perpetuity. It is made available under aCC-BY 4.0 International license.

dCA3, dCA1), **(D)** ventral hippocampal regions (vDG, vCA3, vCA1), and **(E)** amygdalar areas (LA, BLA, BMA, CEA) revealed no difference in neural activity levels between CT and IS groups. **(F-H)** Targeted isocortical analysis showed that eYFP$^+$ cell counts in the RSP and escape latency during later escapable shock testing were significantly correlated in the IS group. **(I-N)** No significant correlations were found between dorsal and ventral hippocampal region eYFP$^+$ cell counts and escape latency during later escapable shock testing. **(O-S)** No significant correlations were found between targeted amygdalar region eYFP cell counts and escape latency. Error bars represent ± SEM. Per brain region analyzed, n = 5 mice for the CT group and n = 5-6 for the IS group. ACA, anterior cingulate area; AI, agranular insula; RSP, retrosplenial area; dDG, dorsal dentate gyrus; dCA3, dorsal CA3; dCA1, dorsal CA1; vDG, ventral dentate gyrus; vCA3, ventral CA3; vCA1, ventral CA1; LA, lateral amygdalar nucleus; BLA, basolateral amygdalar nucleus; BMA, basomedial amygdalar nucleus; CEA, central amygdalar nucleus; MEA, medial amygdalar nucleus.

bioRxiv preprint doi: https://doi.org/10.1101/2024.07.12.603299; this version posted July 16, 2024. The copyright holder for this preprint (which was not certified by peer review) is the author/funder, who has granted bioRxiv a license to display the preprint in perpetuity. It is made available under aCC-BY 4.0 International license.

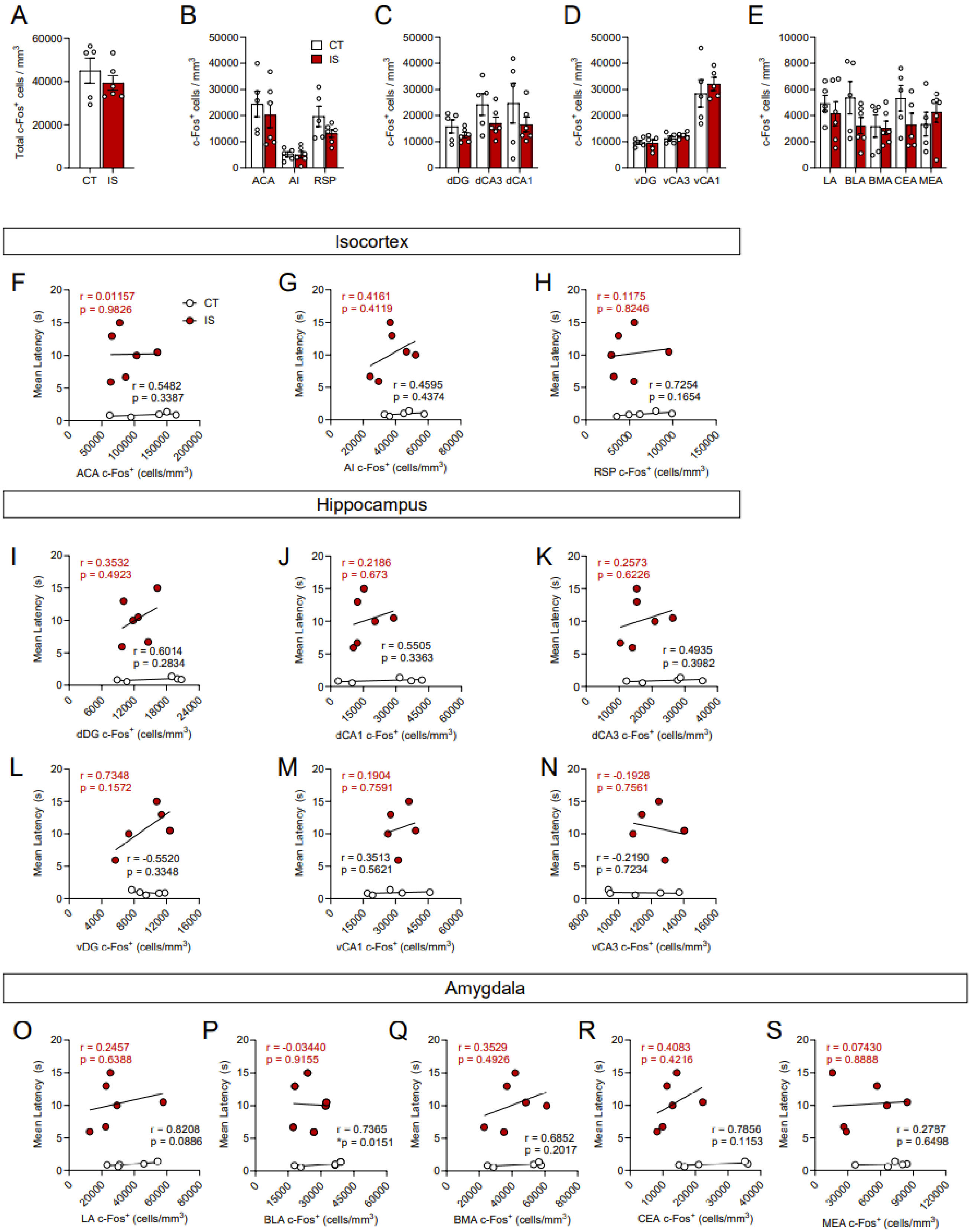

Figure S4. Absolute activity of targeted isocortical, hippocampal, and amygdala regions are not associated with learned helpless expression. (A) No significant differences were found in total brain-wide

bioRxiv preprint doi: https://doi.org/10.1101/2024.07.12.603299; this version posted July 16, 2024. The copyright holder for this preprint (which was not certified by peer review) is the author/funder, who has granted bioRxiv a license to display the preprint in perpetuity. It is made available under aCC-BY 4.0 International license.

volume normalized (cells/mm^3) counts of c-Fos$^+$ cells between context trained (CT) or inescapable shock (IS) groups. Regional activation analysis of targeted **(B)** isocortical areas (ACA, AI, RSP), **(C)** dorsal hippocampal regions (dDG, dCA3, dCA1), **(D)** ventral hippocampal regions (vDG, vCA3, vCA1), and **(E)** amygdalar areas (LA, BLA, BMA, CEA) revealed no difference in neural activity levels between CT and IS groups. **(F-H)** c-Fos$^+$ cell counts in targeted isocortical regions do not correlate with escape latency during later escapable shock. **(I-N)** No significant correlations were found between dorsal and ventral hippocampal region c-Fos$^+$ cell counts and escape latency during later escapable shock testing. **(O-S)** No significant correlations were found between targeted amygdalar region c-Fos$^+$ cell counts and escape latency. Error bars represent ± SEM. Per brain region analyzed, n = 5 mice for the CT group and n = 5-6 for the IS group. ACA, anterior cingulate area; AI, agranular insula; RSP, retrosplenial area; dDG, dorsal dentate gyrus; dCA3, dorsal CA3; dCA1, dorsal CA1; vDG, ventral dentate gyrus; vCA3, ventral CA3; vCA1, ventral CA1; LA, lateral amygdalar nucleus; BLA, basolateral amygdalar nucleus; BMA, basomedial amygdalar nucleus; CEA, central amygdalar nucleus; MEA, medial amygdalar nucleus.

bioRxiv preprint doi: https://doi.org/10.1101/2024.07.12.603299; this version posted July 16, 2024. The copyright holder for this preprint (which was not certified by peer review) is the author/funder, who has granted bioRxiv a license to display the preprint in perpetuity. It is made available under aCC-BY 4.0 International license.

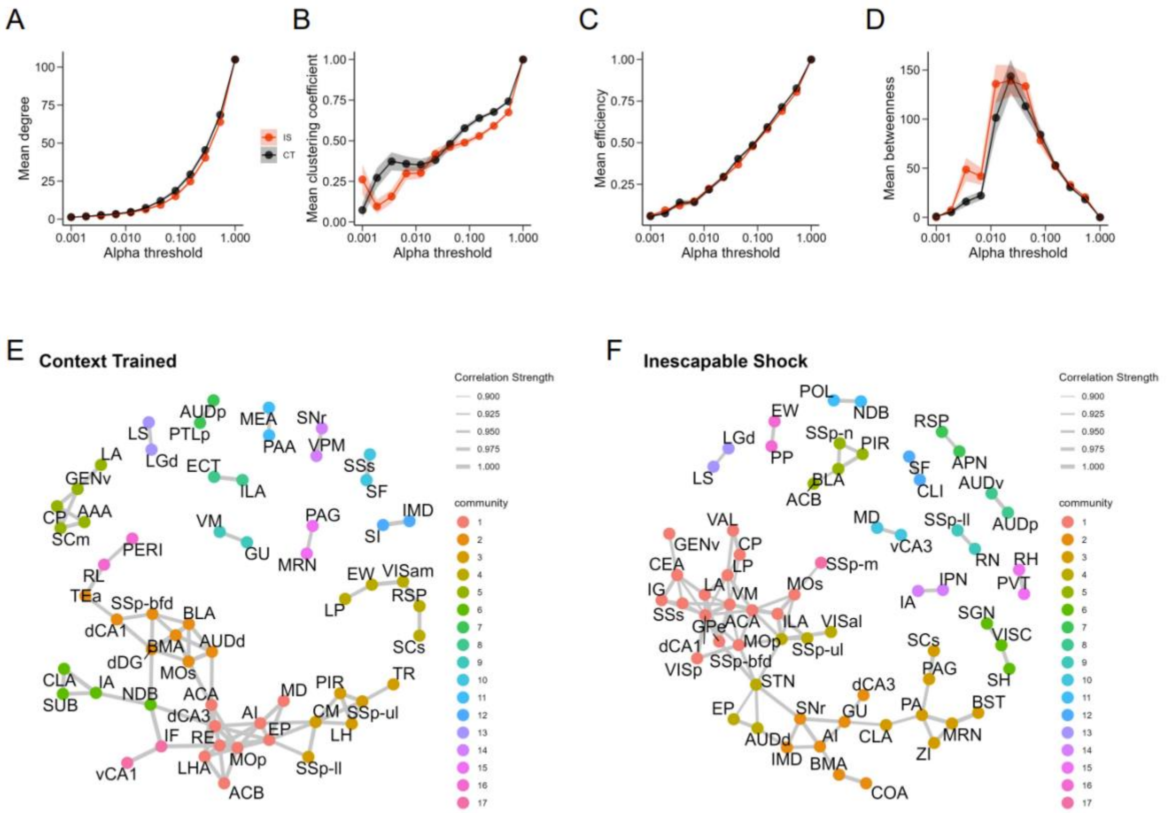

Figure S5. Properties of networks constructed from brain-wide c-Fos+ regional co-expression.

Trajectories of global c-Fos$^+$ network topology metrics of **(A)** mean degree, **(B)** mean clustering coefficient, **(C)** mean efficiency, and **(D)** mean betweenness centrality are calculated based off a wide range of possible significance thresholds. Exploratory community detection analysis applied to **(E)** Context trained (CT) and **(F)** inescapable shock (IS) c-Fos$^+$ networks based on calculation of the leading non-negative eigenvector of the modularity matrix. Gray lines indicate retained network connections between nodes. Nodes belonging to the same detected communities are colored identically. Error shading represents ± SEM; n = 5 mice for the CT group and n = 6 for the IS group.

bioRxiv preprint doi: https://doi.org/10.1101/2024.07.12.603299; this version posted July 16, 2024. The copyright holder for this preprint (which was not certified by peer review) is the author/funder, who has granted bioRxiv a license to display the preprint in perpetuity. It is made available under aCC-BY 4.0 International license.

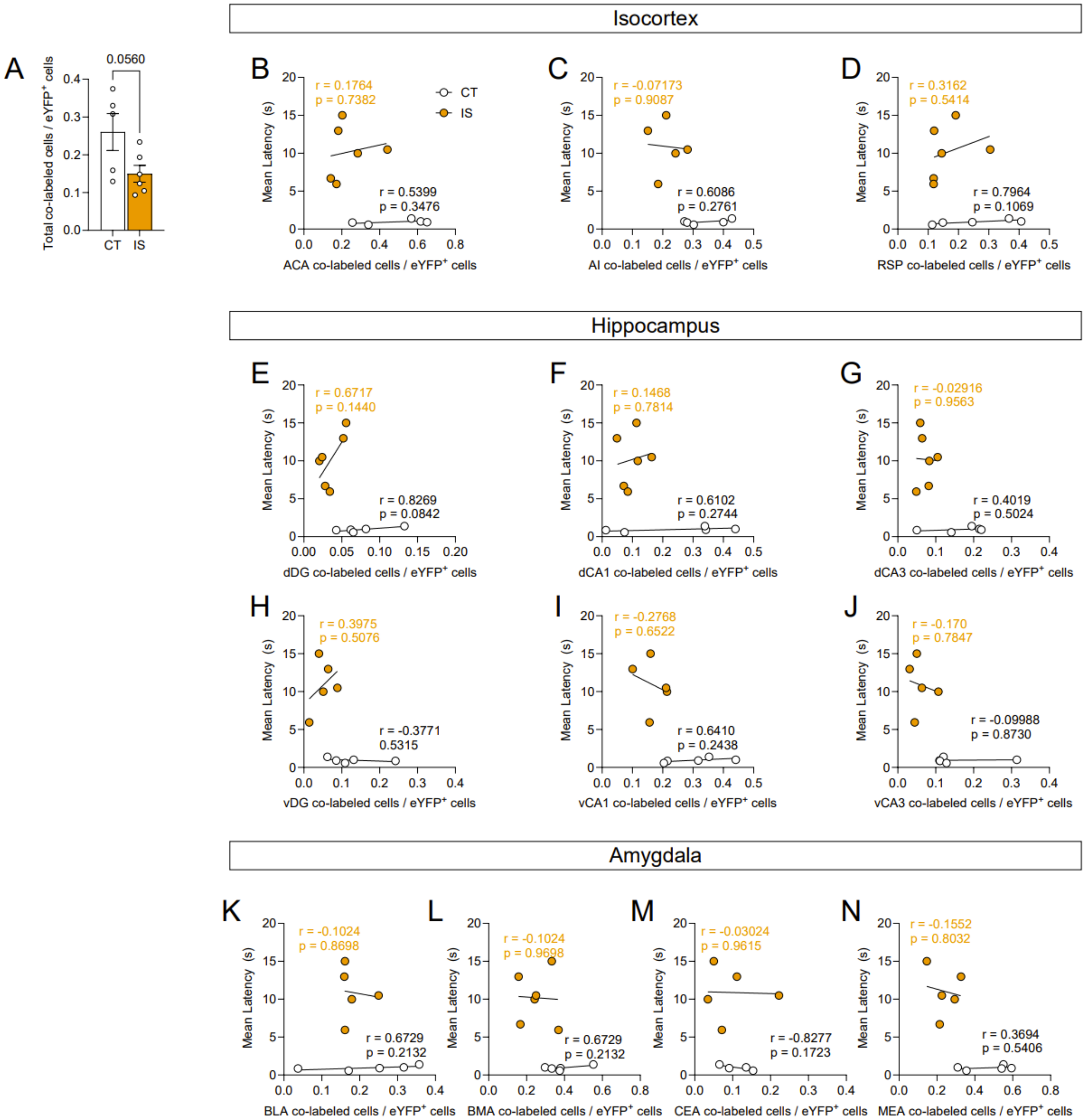

Figure S6. Global reactivation activity during escapable shock is dampened in mice previous exposed to inescapable shock. (A) There is a trending global decrease of proportion of reactivated cells (co-labeled cells / eYFP$^+$ cells) in inescapable shock (IS) mice compared to context trained (CT) mice. Reactivation activity of targeted isocortical areas, such as the **(B)** ACA, **(C)** AI, and **(D)** RSP, is not correlated to escape latency in IS or CT mice. Reactivation activity of targeted dorsal hippocampal regions, such as the **(E)** dDG, **(F)** dCA1, **(G)** and dCA3, and of targeted ventral hippocampal regions, such as the **(H)** vDG, **(I)** vCA1, **(J)** and vCA3, is not correlated to escape latency in IS or CT mice. Reactivation activity of targeted amygdalar areas, such as

bioRxiv preprint doi: https://doi.org/10.1101/2024.07.12.603299; this version posted July 16, 2024. The copyright holder for this preprint (which was not certified by peer review) is the author/funder, who has granted bioRxiv a license to display the preprint in perpetuity. It is made available under aCC-BY 4.0 International license.

the **(K)** BLA, **(L)** BMA, **(M)** CEA, **(N)** and MEA, is not correlated to escape latency in IS or CT mice. Error bars represent ± SEM. Per brain region analyzed, n = 4-5 mice for the CT group and n = 5-6 for the IS group. ACA, anterior cingulate area; AI, agranular insula; RSP, retrosplenial area; dDG, dorsal dentate gyrus; dCA3, dorsal CA3; dCA1, dorsal CA1; vDG , ventral dentate gyrus; vCA3, ventral CA3; vCA1, ventral CA1; LA, lateral amygdalar nucleus; BLA, basolateral amygdalar nucleus; BMA, basomedial amygdalar nucleus; CEA, central amygdalar nucleus; MEA, medial amygdalar nucleus.

bioRxiv preprint doi: https://doi.org/10.1101/2024.07.12.603299; this version posted July 16, 2024. The copyright holder for this preprint (which was not certified by peer review) is the author/funder, who has granted bioRxiv a license to display the preprint in perpetuity. It is made available under aCC-BY 4.0 International license.

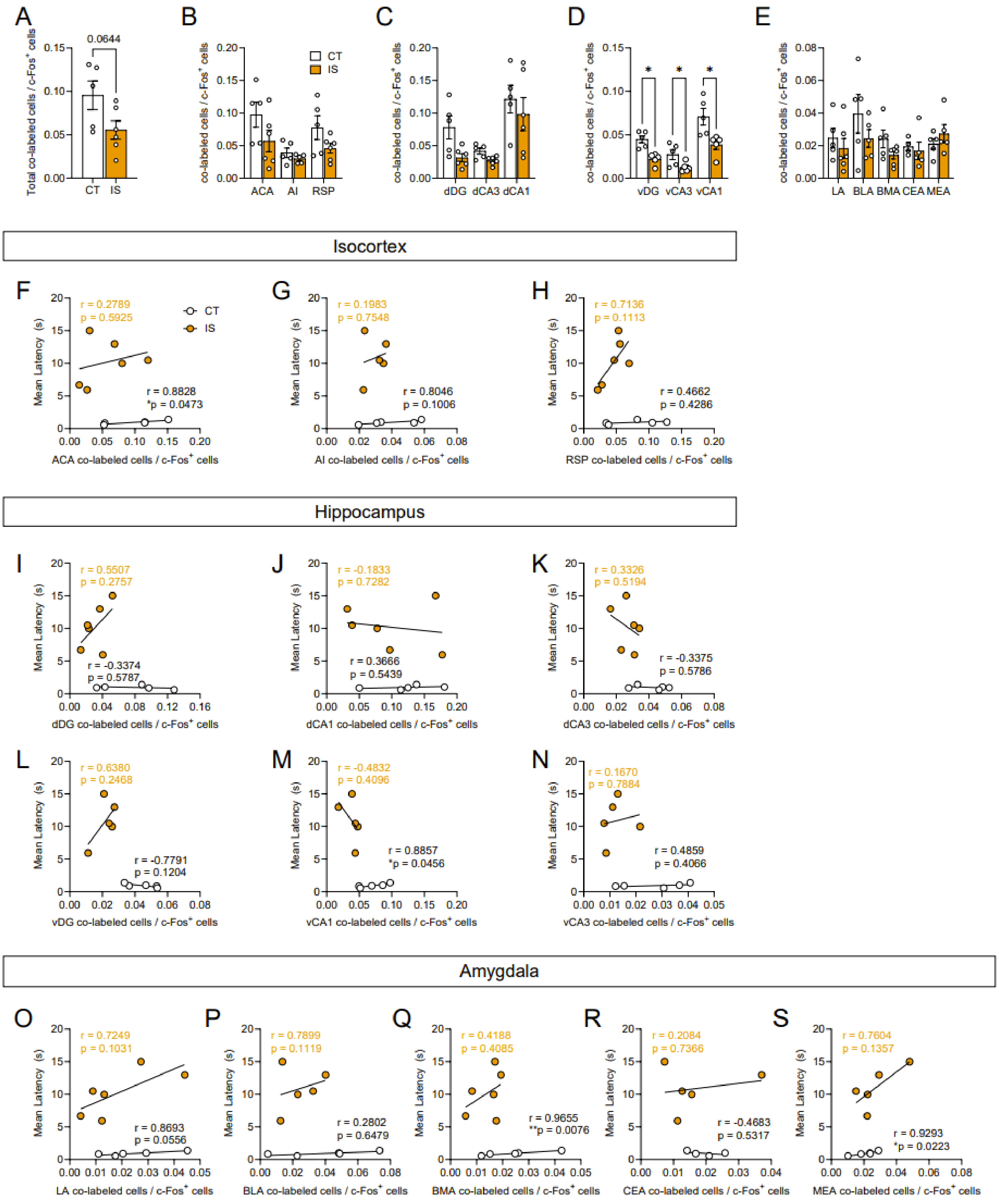

Figure S7. Reactivated activity proportions are decreased in targeted ventral hippocampal regions following inescapable shock. (A) There is a trending decrease in global co-labeled / c-Fos^{+} cell proportions

bioRxiv preprint doi: https://doi.org/10.1101/2024.07.12.603299; this version posted July 16, 2024. The copyright holder for this preprint (which was not certified by peer review) is the author/funder, who has granted bioRxiv a license to display the preprint in perpetuity. It is made available under aCC-BY 4.0 International license.

between context trained (CT) or inescapable shock (IS) groups. Regional activation analysis of targeted **(B)** isocortical areas (ACA, AI, RSP), **(C)** dorsal hippocampal regions (dDG, dCA3, dCA1), **(D)** ventral hippocampal regions (vDG, vCA3, vCA1), and **(E)** amygdalar areas (LA, BLA, BMA, CEA) reveal that vDG, vCA3, and vCA1 activity are significantly decreased in IS mice compared to CT mice. Correlation analysis of co-labeled / c-Fos+ activity of targeted isocortical areas, such as the **(F)** ACA, **(G)** AI, and **(H)** RSP, reveal that ACA activity is significantly correlated with crossing latency in the CT group. Analysis of targeted dorsal and ventral hippocampal regions, such as the **(I)** dDG, **(J)** dCA1, **(K)** dCA3, **(L)** vDG, **(M)** vCA1, **(N)** and vCA3, reveal that reveal that vCA1 co-labeled / c-Fos+ is significantly correlated with crossing latency in the CT group. Analysis of targeted amygdalar regions, such as **(O)** LA, **(P)** BLA, **(Q)** BMA, **(R)** CEA, **(S)** MEA reveal that reveal that co-labeled / c-Fos+ activity in the BMA and MEA is significantly correlated with crossing latency in the CT group. Error bars represent ± SEM. Per brain region, n = 4-5 mice for the CT group and n = 5-6 for the IS group. ACA, anterior cingulate area; AI, agranular insula; RSP, retrosplenial area; dDG, dorsal dentate gyrus; dCA3, dorsal CA3; dCA1, dorsal CA1; vDG, ventral dentate gyrus; vCA3, ventral CA3; vCA1, ventral CA1; LA, lateral amygdalar nucleus; BLA, basolateral amygdalar nucleus; BMA, basomedial amygdalar nucleus; CEA, central amygdalar nucleus; MEA, medial amygdalar nucleus.

bioRxiv preprint doi: https://doi.org/10.1101/2024.07.12.603299; this version posted July 16, 2024. The copyright holder for this preprint (which was not certified by peer review) is the author/funder, who has granted bioRxiv a license to display the preprint in perpetuity. It is made available under aCC-BY 4.0 International license.

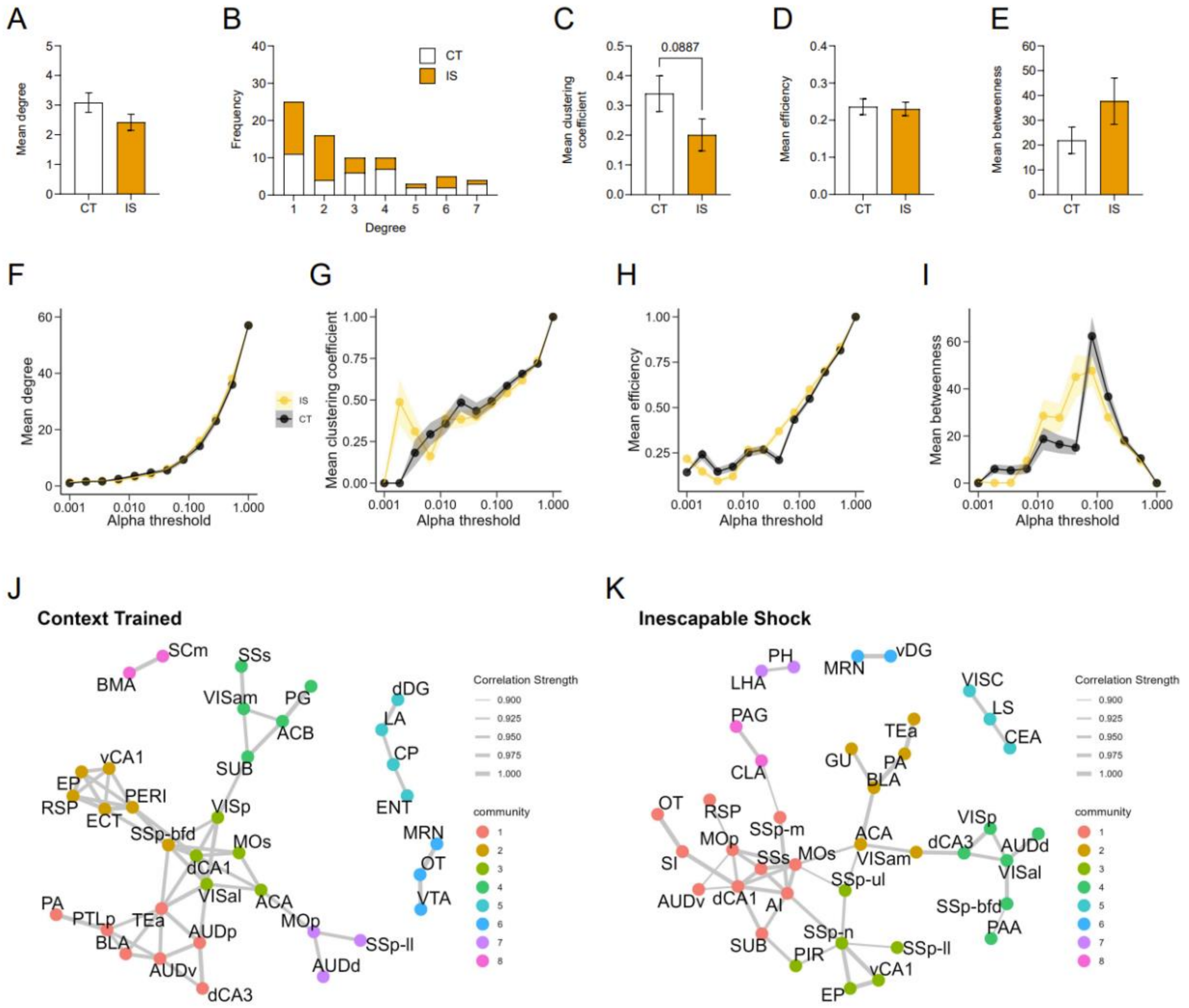

Figure S8. Properties of networks constructed from functional connections between reactivated ensembles (co-labeled / eYFP⁺ cells). Global topology characteristics of CT and IS reactivation networks were calculated after thresholding for important regional correlations using an r > 0.9 and p < 0.01. (**A**) Average degree does not differ between groups. (**B**) Degree frequency distribution shows a right-tailed distribution for both groups. (**C-E**) Mean clustering coefficient, global efficiency, and mean betweenness centrality does not differ between groups. Trajectories of global network topology metrics, including **(F)** mean degree, **(G)** mean clustering coefficient, **(H)** mean efficiency, and **(I)** mean betweenness, were calculated based off a wide range of possible significance thresholds. Exploratory community detection analysis applied to **(J)** CT and **(K)** IS reactivation networks based on calculation of the leading non-negative eigenvector of the modularity matrix. Gray lines indicate retained network connections between nodes. Nodes belonging to the

bioRxiv preprint doi: https://doi.org/10.1101/2024.07.12.603299; this version posted July 16, 2024. The copyright holder for this preprint (which was not certified by peer review) is the author/funder, who has granted bioRxiv a license to display the preprint in perpetuity. It is made available under aCC-BY 4.0 International license.

same detected communities are identically colored. Error bars represent ± SEM; n = 5 mice for the CT group and n = 6 for the IS group.

bioRxiv preprint doi: https://doi.org/10.1101/2024.07.12.603299; this version posted July 16, 2024. The copyright holder for this preprint (which was not certified by peer review) is the author/funder, who has granted bioRxiv a license to display the preprint in perpetuity. It is made available under aCC-BY 4.0 International license.

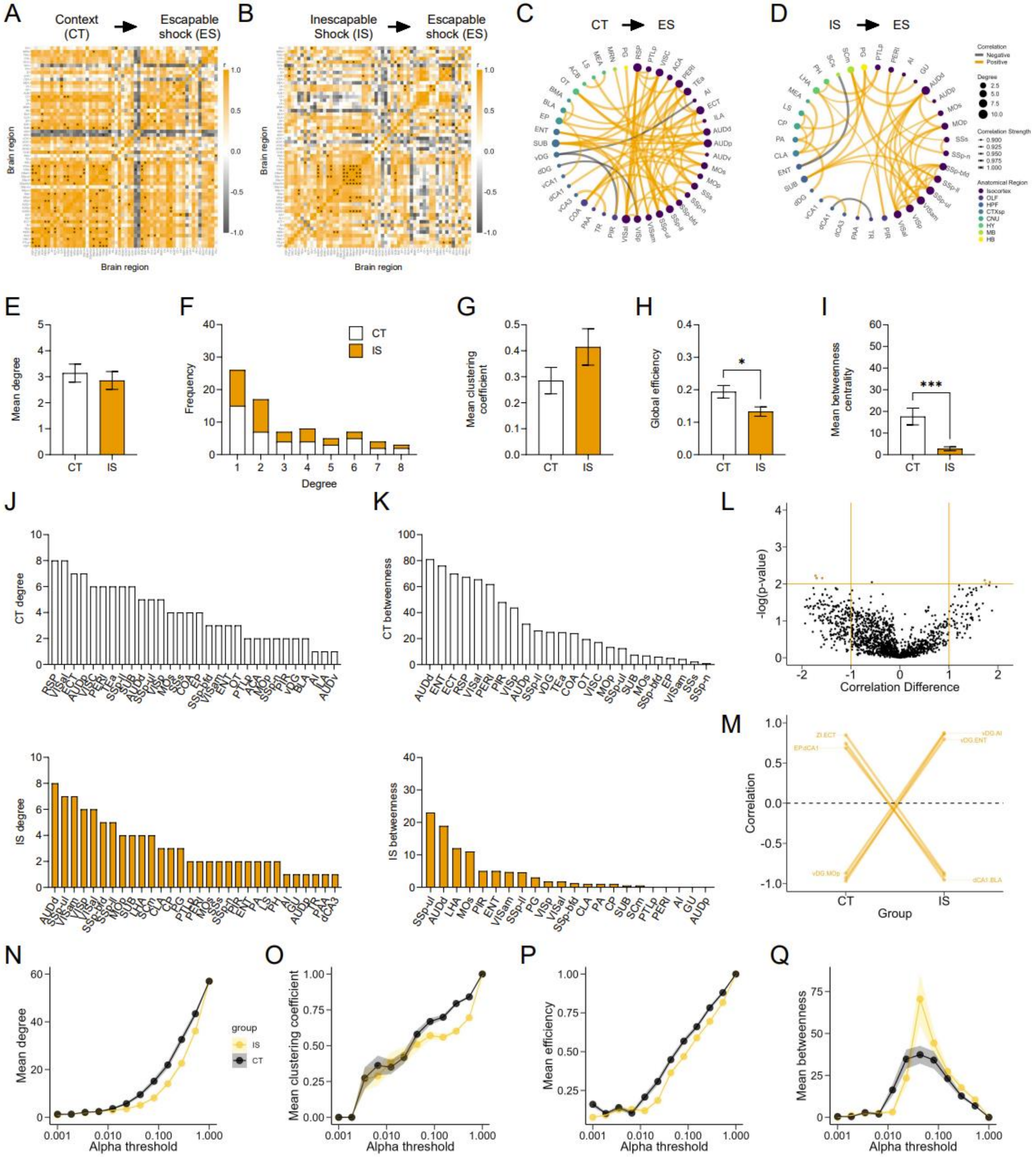

Figure S9. Supplementary network level analysis of co-labeled / c-Fos$^+$ activity reveals high sensory functional connectivity. (A-B) Regional cross correlation heatmaps of colabel / c-Fos$^+$ proportions in context

bioRxiv preprint doi: https://doi.org/10.1101/2024.07.12.603299; this version posted July 16, 2024. The copyright holder for this preprint (which was not certified by peer review) is the author/funder, who has granted bioRxiv a license to display the preprint in perpetuity. It is made available under aCC-BY 4.0 International license.

trained (CT) and learned helpless (IS) mice. Significant values are $p < 0.01$. In the IS heatmaps, an especially high co-activation amongst somatosensory regions, visual, and auditory regions is observed. (**C-D**) Functional networks constructed after thresholding for the strongest and most significantly correlated or anti-correlated connections ($r > 0.9$, $p < 0.01$). Average degree centrality does not differ between IS and CT groups. (**F**) Degree frequency distributions are right-tailed. (**G-H**) Mean clustering coefficient and global efficiency do not differ between the CT and IS networks. (**I**) Mean betweenness centrality is significantly lower in IS networks, likely due to overall lower interconnectivity amongst regions. (**J-K**) The top node degree values in descending order for the CT (white, top) and IS (yellow, bottom) networks indicate which regions are most highly connected. (**L-M**) The top node betweenness values in descending order for the CT (white, top) and IS (yellow, bottom) networks. (**N**) Volcano plot of the Pearson correlation differences ($r_{IS} – r_{CT}$) for all individual regional connections against their p-values calculated from a permutation analysis. Points intersecting or within the upper left or right quadrant represent the regional relationships with the greatest change (|correlation difference| > 1), that were most significant ($p < 0.01$). (**O**) A parallel coordinate plot highlighting individual significantly changed regional correlations between groups, as well as the direction of their change. **(P-S)** Trajectories of global c-Fos$^+$ network topology metrics of mean degree, mean efficiency, mean betweenness centrality, and mean clustering coefficients are calculated based off a wide range of possible significance thresholds. ***$p < 0.001$. Error bars represent ± SEM; n = 5 mice for the CT group and n = 6 for the IS group.

bioRxiv preprint doi: https://doi.org/10.1101/2024.07.12.603299; this version posted July 16, 2024. The copyright holder for this preprint (which was not certified by peer review) is the author/funder, who has granted bioRxiv a license to display the preprint in perpetuity. It is made available under aCC-BY 4.0 International license.

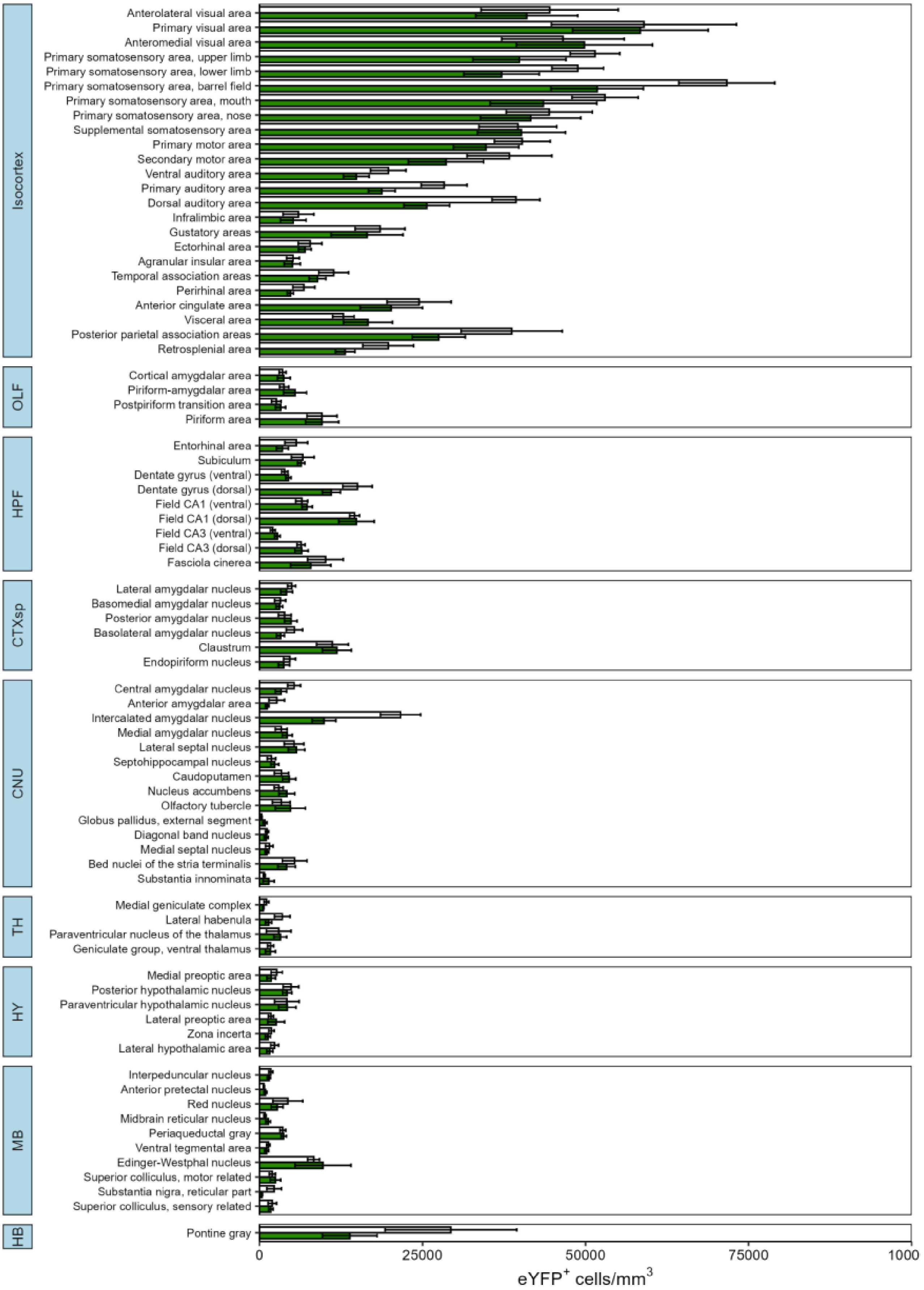

bioRxiv preprint doi: https://doi.org/10.1101/2024.07.12.603299; this version posted July 16, 2024. The copyright holder for this preprint (which was not certified by peer review) is the author/funder, who has granted bioRxiv a license to display the preprint in perpetuity. It is made available under aCC-BY 4.0 International license.

Figure S10. Differential expression of eYFP activity across all mapped regions. Volume normalized (cells/mm^3) counts of eYFP$^+$ cells between context trained (CT, white bars) or inescapable shock (IS, green bars) mice across all regions mapped. Subregion activity expression patterns are organized by parent anatomical divisions. Error bars represent ± SEM. OLF, olfactory areas; HPF, hippocampal formation; CTXsp, cortical subplate; CNU, cerebral nuclei; TH, thalamus; HY, hypothalamus; MB, midbrain; HB, hindbrain. Error bars represent ± SEM; n = 5 mice for the CT group and n = 6 for the IS group.

bioRxiv preprint doi: https://doi.org/10.1101/2024.07.12.603299; this version posted July 16, 2024. The copyright holder for this preprint (which was not certified by peer review) is the author/funder, who has granted bioRxiv a license to display the preprint in perpetuity. It is made available under aCC-BY 4.0 International license.

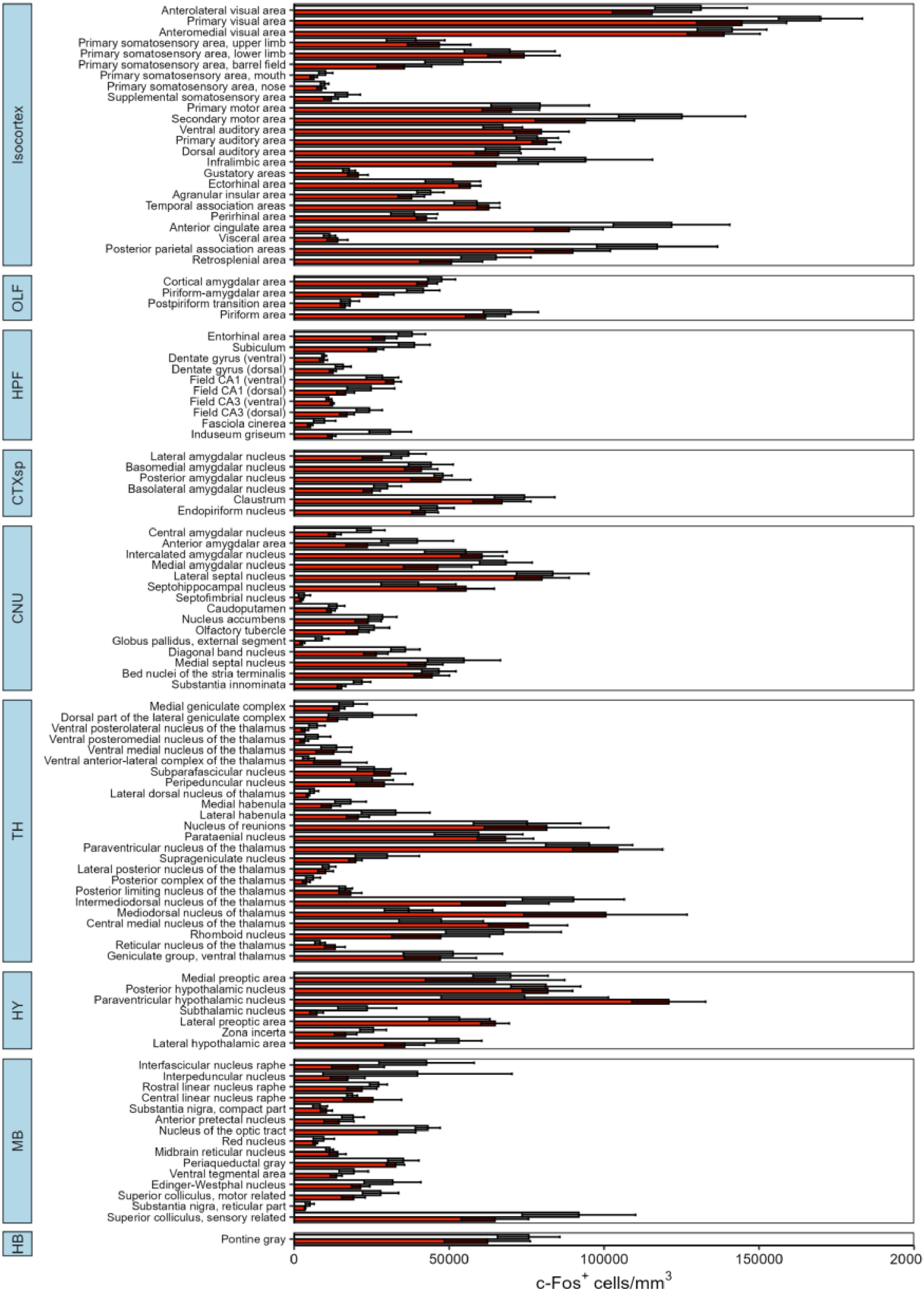

bioRxiv preprint doi: https://doi.org/10.1101/2024.07.12.603299; this version posted July 16, 2024. The copyright holder for this preprint (which was not certified by peer review) is the author/funder, who has granted bioRxiv a license to display the preprint in perpetuity. It is made available under aCC-BY 4.0 International license.

Figure S11. Differential expression of c-Fos activity across all mapped regions. Volume normalized (cells/mm^3) counts of c-Fos$^+$ cells between context trained (CT, white bars) or inescapable shock (IS, red bars) mice across all regions mapped. Subregion activity expression patterns are organized by parent anatomical divisions. Error bars represent ± SEM. OLF, olfactory areas; HPF, hippocampal formation; CTXsp, cortical subplate; CNU, cerebral nuclei; TH, thalamus; HY, hypothalamus; MB, midbrain; HB, hindbrain. Error bars represent ± SEM; n = 5 mice for the CT group and n = 6 for the IS group.

bioRxiv preprint doi: https://doi.org/10.1101/2024.07.12.603299; this version posted July 16, 2024. The copyright holder for this preprint (which was not certified by peer review) is the author/funder, who has granted bioRxiv a license to display the preprint in perpetuity. It is made available under aCC-BY 4.0 International license.

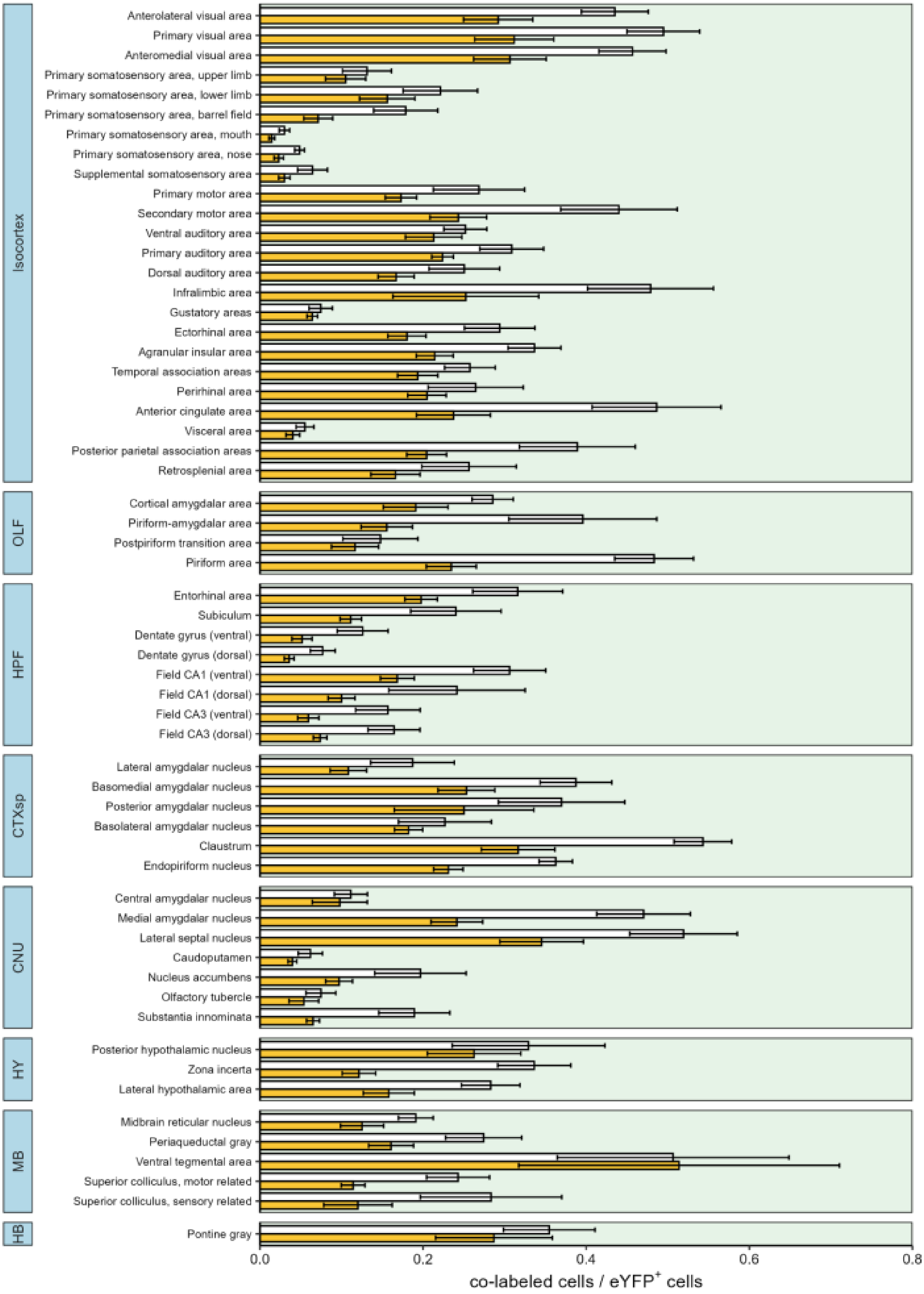

bioRxiv preprint doi: https://doi.org/10.1101/2024.07.12.603299; this version posted July 16, 2024. The copyright holder for this preprint (which was not certified by peer review) is the author/funder, who has granted bioRxiv a license to display the preprint in perpetuity. It is made available under aCC-BY 4.0 International license.

Figure S12. Differential reactivation proportions across all mapped regions. Reactivation activity (colabelled cells / eYFP$^+$ cells) between context trained (CT, white bars) or inescapable shock (IS, yellow bars) mice across all regions mapped. Subregion activity expression patterns are organized by parent anatomical divisions. Error bars represent ± SEM. OLF, olfactory areas; HPF, hippocampal formation; CTXsp, cortical subplate; CNU, cerebral nuclei; TH, thalamus; HY, hypothalamus; MB, midbrain; HB, hindbrain. Error bars represent ± SEM; n = 5 mice for the CT group and n = 6 for the IS group.

bioRxiv preprint doi: https://doi.org/10.1101/2024.07.12.603299; this version posted July 16, 2024. The copyright holder for this preprint (which was not certified by peer review) is the author/funder, who has granted bioRxiv a license to display the preprint in perpetuity. It is made available under aCC-BY 4.0 International license.

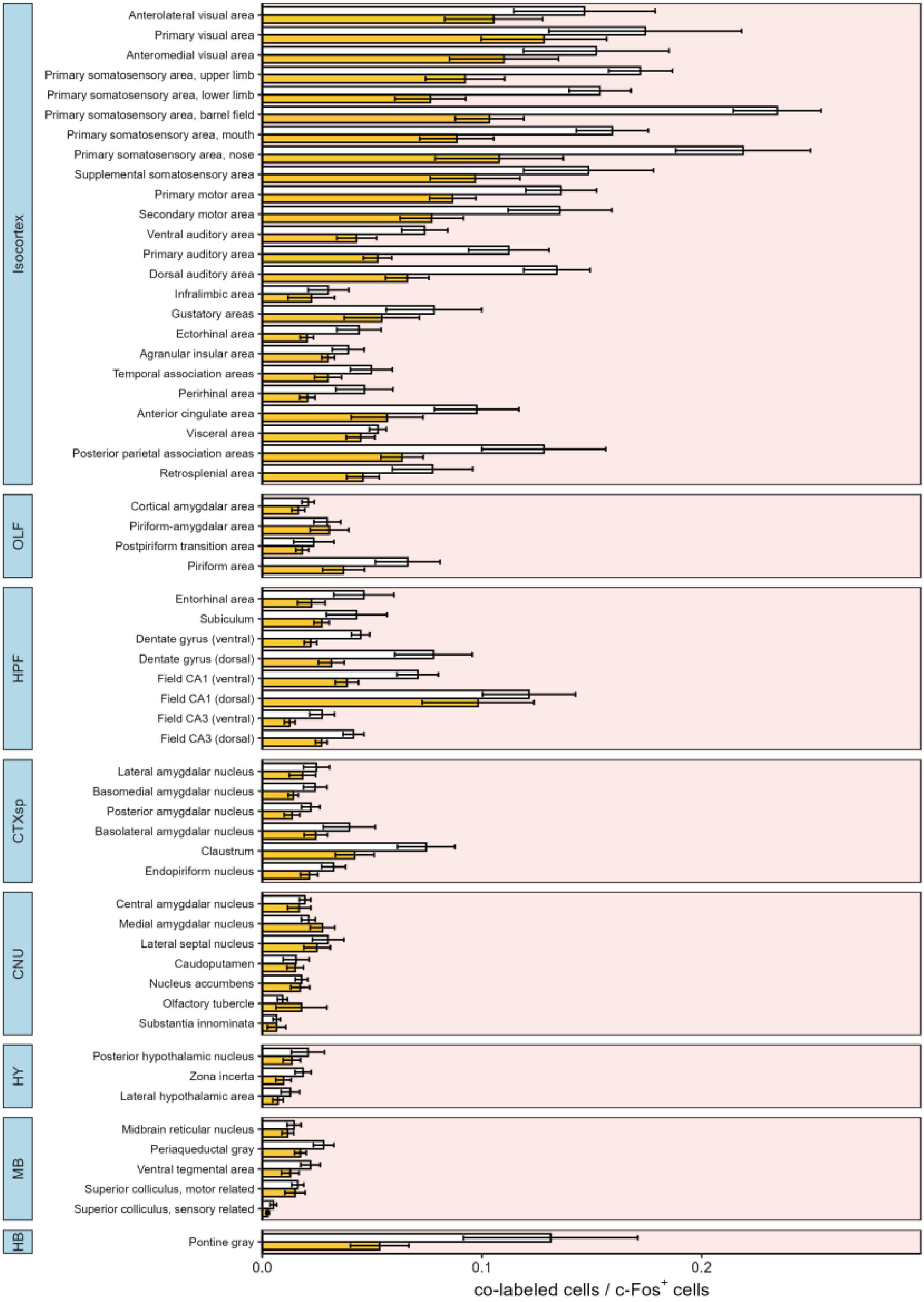

bioRxiv preprint doi: https://doi.org/10.1101/2024.07.12.603299; this version posted July 16, 2024. The copyright holder for this preprint (which was not certified by peer review) is the author/funder, who has granted bioRxiv a license to display the preprint in perpetuity. It is made available under aCC-BY 4.0 International license.

Figure S13. Differential co-labeled / c-Fos$^+$ proportions across all mapped regions. Co-labeled/c-Fos$^+$ proportions between context trained (CT, white bars) or inescapable shock (IS, yellow bars) mice across all regions mapped. Subregion activity expression patterns are organized by parent anatomical divisions. Error bars represent ± SEM. OLF, olfactory areas; HPF, hippocampal formation; CTXsp, cortical subplate; CNU, cerebral nuclei; TH, thalamus; HY, hypothalamus; MB, midbrain; HB, hindbrain. Error bars represent ± SEM; n = 5 mice for the CT group and n = 6 for the IS group.

bioRxiv preprint doi: https://doi.org/10.1101/2024.07.12.603299; this version posted July 16, 2024. The copyright holder for this preprint (which was not certified by peer review) is the author/funder, who has granted bioRxiv a license to display the preprint in perpetuity. It is made available under aCC-BY 4.0 International license.

SUPPLEMENTAL TABLE LEGENDS

Table S1. Key resources summary.

Table S2. Statistical analysis summary for all behavioral tests.

Table S3. Comprehensive list of acronyms used.

Table S4. Statistical analysis summary for targeted brain region analyses.

Table S5. Statistical analysis summary for eYFP activity across all mapped regions. False discovery rate correction of p-values can be found under the p.adj column.

Table S6. Statistical analysis summary for c-Fos activity across all mapped regions. False discovery rate correction of p-values can be found under the p.adj column.

Table S7. Statistical analysis summary for reactivation proportion (co-labeled cells / eYFP$^+$ cells) across all mapped regions. False discovery rate correction of p-values can be found under the p.adj column.

Table S8. Statistical analysis summary for proportion of reactivation activity (co-labeled cells / c-Fos$^+$ cells) across all mapped regions. False discovery rate correction of p-values can be found under the p.adj column.
